# Eco-Physiological Responses of Dominant Species to Watering in a Natural Grassland Community on the Semi-Arid Loess Plateau of China

**DOI:** 10.3389/fpls.2016.00663

**Published:** 2016-05-18

**Authors:** Furong Niu, Dongping Duan, Ji Chen, Peifeng Xiong, He Zhang, Zhi Wang, Bingcheng Xu

**Affiliations:** ^1^State Key Laboratory of Soil Erosion and Dryland Farming on the Loess Plateau, Northwest A&F UniversityYangling, China; ^2^Institute of Soil and Water Conservation, Chinese Academy of Sciences and Ministry of Water ResourcesYangling, China

**Keywords:** *Bothriochloa ischaemum*, *Lespedeza davurica*, photosynthetic parameters, community biomass, precipitation regime

## Abstract

Altered precipitation regimes significantly affect ecosystem structure and function in arid and semi-arid regions. In order to investigate effects of precipitation changes on natural grassland community in the semi-arid Loess Plateau, the current research examined eco-physiological characteristics of two co-dominant species (i.e., *Bothriochloa ischaemum* and *Lespedeza davurica*) and community composition following two watering instances (i.e., precipitation pulses, July and August, 2011, respectively) in a natural grassland community. Results showed that the photosynthetic rate, transpiration rate, stomatal conductance and intercellular CO_2_ concentration rapidly increased on the first to third day following watering in both species, and both months. Under watering treatments, the maximum net photosynthetic rates appeared on the second to third day after watering, which increased 30–80% in *B. ischaemum* and 40–50% in *L. davurica* compared with non-watering treatments, respectively. Leaf water use efficiency kept stable or initially decreased in both species under watering treatments. Watering in July produced more promoting effects on grass photosynthesis than in August, particularly in *B. ischaemum*. Community above-ground biomass at the end of the growing season increased after watering, although no significant changes in species diversity were observed. Our results indicated that timing and magnitude of watering could significantly affect plant eco-physiological processes, and there were species-specific responses in *B. ischaemum* and *L. davurica*. Pulsed watering increased community productivity, while did not significantly alter community composition after one growing season. The outcomes of this study highlight eco-physiological traits in dominant species may playing important roles in reshaping community composition under altered precipitation regimes.

## Introduction

There is substantial observational evidence which shows that natural ecosystems are being affected in recent decades by climate changes, including rising temperature and altered precipitation regimes (IPCC, 2007). Precipitation is directly linked with water availability, and is regarded as one of the most important factors that affect plant physiological processes in individual level and structure and function in ecosystem scale (Knapp et al., 2001; Pennington and Collins, 2007; Suttle et al., 2007; Thomey et al., 2014). In arid and semi-arid environments, precipitation usually falls as discrete, episodic and unpredictable pulse events, and can greatly affect plant growth, species distribution and community composition (Noy-Meir, 1973; Beier et al., 2012; Sala et al., 2015). Responses of terrestrial ecosystems and their component species to fluctuating precipitation regimes are frequently reported, covering from plant eco-physiological characteristics to ecosystem structure and functions (Schwinning and Sala, 2004; Xu and Li, 2006; Xu et al., 2007; Bai et al., 2008; Heisler-White et al., 2008, 2009; Zeppel et al., 2014). The results showed that neutral and positive photosynthetic responses of grasses and shrubs (Huxman et al., 2004; Xu and Li, 2006; Ignace et al., 2007; Loik, 2007; Throop et al., 2012; Padilla et al., 2013; Thomey et al., 2014), shifts in community composition (Báez et al., 2012; Hoeppner and Dukes, 2012; Prevéy and Seastedt, 2014), and increased plant biomass and ecosystem productivity following precipitation changes in arid and semi-arid ecosystems (Knapp et al., 2001; Bai et al., 2008; Heisler-White et al., 2008; Hoeppner and Dukes, 2012; Koerner et al., 2014; Wilcox et al., 2015).

General Circulation Models have projected that more frequent and intense precipitation events, and longer drought duration might appear in the Loess Plateau region (Li et al., 2012), which would be expected to impact growth and distribution of dominant species, and composition and functions of natural grassland community (Shi et al., 2011; Fan et al., 2012; Chen et al., 2014). The Loess Plateau, located in upper-middle reaches of the Yellow River in northern China, is well-known for its hilly loess terrains and serious soil erosion, which are largely caused by over-cultivations of marginal lands and destruction of natural vegetation (Lu and van Ittersum, 2004). The intense agricultural activities combined with water limitation induced by low annual precipitation, have led to serious ecosystem degradation. Natural grassland accounts for 30–40% of the total land area in the semi-arid loess hilly-gully region, and is the most extensively distributed natural vegetation type (Chen and Wan, 2002). *Bothriochloa ischaemum* (L.) Keng (a perennial C_4_ herbaceous grass) and *Lespedeza davurica* (Laxm.) Schindl. (a perennial C_3_ leguminous sub-shrub) are two co-dominant species in the local natural grassland community, and both play important roles in reducing soil and water loss, and maintaining ecological functions (Xu et al., 2011). Previous studies showed that water infiltration after precipitation events can affect soil depth to 200 cm in the region, and in natural grass it has a rapid infiltration rate and could reach a greater depth than in crops or other vegetation types (e.g., trees, sub-shrubs, and shrubs; Chen et al., 2008; Wang et al., 2013), which imply that grassland may has more sensitive responses to precipitation pulses among all vegetation types in the region. While to our knowledge, responses of semi-arid grassland to precipitation pulses in the Losses Plateau have not yet been systematically reported.

Plants adapt to given environmental conditions by using different morphological and physiological traits, e.g., plant size, plant architecture, rooting depth, water use strategies, and photosynthetic pathways (Meinzer, 2003; McCulley et al., 2005; Volder et al., 2010; Throop et al., 2012). In water-limited regions, short-term precipitation fluctuations rather than annual precipitation amount have more effects on ecosystem structure and function (Huxman et al., 2004; Schwinning and Sala, 2004; Bai et al., 2008). Differences of eco-physiological traits at specie-level are closely correlated with population dynamics, which are important parameters for predicting responses of plant individuals and shifts in the community to fluctuated water sources (Robinson and Gross, 2010). To fully clarify eco-physiological responses of the two co-dominant species to precipitation pulses, we conducted a manipulation experiment in form of intermittent water addition on a natural grassland community, and observed changes of photosynthetic parameters in following days after watering. Our objective was to investigate and compare photosynthetic responses of two co-dominant species after precipitation pulses. Additionally, community biomass and community composition were studied after one growing season.

## Materials and methods

### Site description

The experiment was conducted at the Ansai Research Station of the Chinese Academy of Science (36°51′ N, 109°19′ E, and elevation ranges from 1068 to 1309 m a.s.l.). The Ansai Research Station is located in Ansai County, Shaanxi Province, China, which is characterized by the semi-arid, forest-steppe region on the Loess Plateau. Mean annual precipitation is 540 mm, and precipitation from April to October accounts for 85–95% of the annual total. Precipitation during July to September accounts for 60–80%, and thus the period is normally called the rainy season (Shan and Chen, 1993). Average annual temperature is 8.8°C, with the lowest of −6.9°C in January and the highest of 22.6°C in July. The soil type is classified as Calcic Cambisols that developed on wind-deposited loessic parent material (Wang et al., 2003). The investigated natural grassland communities were distributed in homogeneous vegetation (36°51′ N, 109°18′ E, and elevation 1200 m a.s.l.), with slope gradient and aspect of the experimental area are 20° and SE 15°, respectively. *B. ischaemum* and *L. davurica* represent co-dominant species within the natural grassland community, and other main species include *Artemisia vestita, Astragalus melilotoides, Cleistogenes chinensis, Stipa bungeana* and *Leymus secalinus*.

### Watering treatment

Before water addition, antecedent 0–100 cm average soil water content (θ) was measured by soil drill (Ø4 cm cores) method at each 10 cm depth (oven drying at 105°C to constant weight). Then, in accordance with initial soil water content (θ), irrigated water quantities were calculated as 0.1 × θ (W1) and 0.2 × θ (W2), and corresponding precipitation amounts were calculated by precipitation (mm) = water quantity × *H* (soil depth, 100 cm) × ρb (soil bulk density, 1.2 g cm^−3^; Table 1).

**Table 1:** **Water addition and corresponding precipitation amounts under each treatment**.

**Treatment**	**Date**	**Watering quantity**	**Antecedent soil water content (θ; %)**	**Corresponding precipitation amount (mm)**
M1W1	9 July, 2011	0.1 × θ	7.8	9.4
M1W2		0.2 × θ		18.8
M2W1	7 August, 2011	0.1 × θ	7.6	9.1
M2W2		0.2 × θ		18.2
M3W1	Both dates	0.1 × θ	7.8 and 7.6	18.5
M3W2		0.2 × θ		37.0
CK	–	–	–	–

*θ means average soil water content of 0–100 cm soil depth in the grassland before watering; “M1” means watering on 9 July, 2011; “M2” means watering on 7 August, 2011; “M3” means watering on both 9 July and 7 August, 2011; “W1” means watering quantities were 0.1 × θ; “W2” means watering quantities were 0.2 × θ; “M” and “W” combinations stand for different watering treatments, “CK” stands for control treatment with no water addition*.

Watering treatments (i.e., precipitation pulses) were carried out in 2011. Two watering events were taken place on 9 July (M1) and 7 August (M2), respectively. Treatments received watering on both dates represent by “M3.” Combinations of watering date (M) and quantity (W) stand for 6 watering and one control treatments (see Table 1). Each treatment was replicated three times, and thus totally 21 plots were randomly set up across the experimental area (ca. 0.3 ha). Each plot measured 2 × 2 m and was situated at least 2 m apart from each other to avoid potential neighboring influence. Water was slowly and evenly irrigated by sprinklers from community ground to avoid runoff and plant interception loss at 18:00 PM on each date.

### Photosynthesis measurement

One youngest fully expanded and healthy leaf of each of two species (i.e., *B. ischaemum* and *L. davurica*) per plot was chosen for performing photosynthetic measurements, and this leaf was measured on 1 day before watering and consecutive days after watering, i.e., the first to seventh day in July and the first to fifth day in August. Photosynthetic measurements were conducted at 9:00–11:00 AM to capture instantaneous values of photosynthetic parameters by using a portable photosynthesis system (CIRAS-2; PP SYSTEMS, Haverhill, MA, USA). During measurements, light intensities and concentrations of CO_2_ were set to ambient conditions, which were 349 ± 3.3 μmol mol^−1^ (mean ± SE) and 2110 ± 10.4 μmol m^−2^ s^−1^, respectively; air humidity (60%) and air flow (200 μmol m^−2^ s^−1^) were controlled by the portable photosynthesis system. The measured photosynthetic parameters included the net photosynthetic rate (*P*_n_; μmol m^−2^ s^−1^), transpiration rate (*E*; mmol m^−2^ s^−1^), stomatal conductance (*g*_s_; mmol m^−2^ s^−1^), and intercellular CO_2_ concentration (*C*_i_; μmol mol^−1^). Leaf instantaneous water use efficiency (WUE; mmol mol^−1^) was calculated by dividing *P*_n_ by *E*.

### Environmental factors

Annual precipitation data for 2011 were sourced from the meteorological station located close to the study site (ca. 250 m). Instantaneous values of photosynthetically active radiation (PAR; μmol m^−2^ s^−1^), air temperature (*T*_a_; °C) and air relative humidity (RH; %) were recorded by the portable photosynthesis system between 9:00 and 11:00 AM on each day, and were used to represent micro-meteorological conditions of the experimental area during the experimental period.

### Community characteristics

One 1 × 1 m quadrat from each plot was randomly chosen for above-ground biomass measurement at the beginning (on 15 June) and end of the growing season (on 2 November). In addition, community coverage, species number and density in each plot were assessed on the two dates. The biomass production was dried at 80°C for 48 h.

The species richness (*R*) was defined as average species number across three replicated plots under the same treatment. The importance value index (IVI) was employed to evaluate species ecological importance; it was calculated as:

$$\begin{array}{l} {IVI}_{i}={RF}_{i}+{RD}_{i}+{RC}_{i} \end{array}$$

Where *RF*_*i*_ is the relative frequency, which calculated as the frequency of species *i* divided by the total frequency of the plot; *RD*_*i*_ is the relative density, which calculated as the density of species *i* divided by the total density of the plot; *RC*_*i*_ is the relative coverage, which calculated as the coverage of species *i* divided by the total coverage of the plot.

The Shannon-Wiener diversity index (*H*) was calculated as:

$$\begin{array}{l} H=-\sum P_{i}\times \text{ln }P_{i}\; \end{array}$$

Where *P*_*i*_ is the relative abundance of species *i*.

The Simpson's diversity index (*D*) was calculated as:

$$\begin{array}{l} D=\sum P_{i}^{2} \end{array}$$

The Pielou's evenness index (*J*) was calculated as:

$$\begin{array}{l} J=H/\text{ln }N\; \end{array}$$

Where *N* is the total number of species.

All species diversity and evenness indices were calculated following Magurran (2004).

### Statistical analysis

One-way analyses of variance (ANOVA) were used to compare differences between 5-day-averaged net photosynthetic rates after watering and community above-ground biomasses at the end of the growing season throughout different treatments. Tukey's honestly significant difference (HSD) tests were performed to identify significant different groups. Prior to ANOVA, data were checked for normality (Shapiro-Wilk test) and homogeneity of variances (Levene's test). Kruskal-Wallis tests were used to compare differences of species diversity indices and importance value index (IVI) between treatments due to normal distributions of variables could not be obtained by data transformations. All statistical analyses were performed with SPSS 17.0 (SPSS Inc., Chicago, IL, USA). A *P* < 0.05 was considered statistically significant.

## Results

### Environmental factors

**Figure 1:**
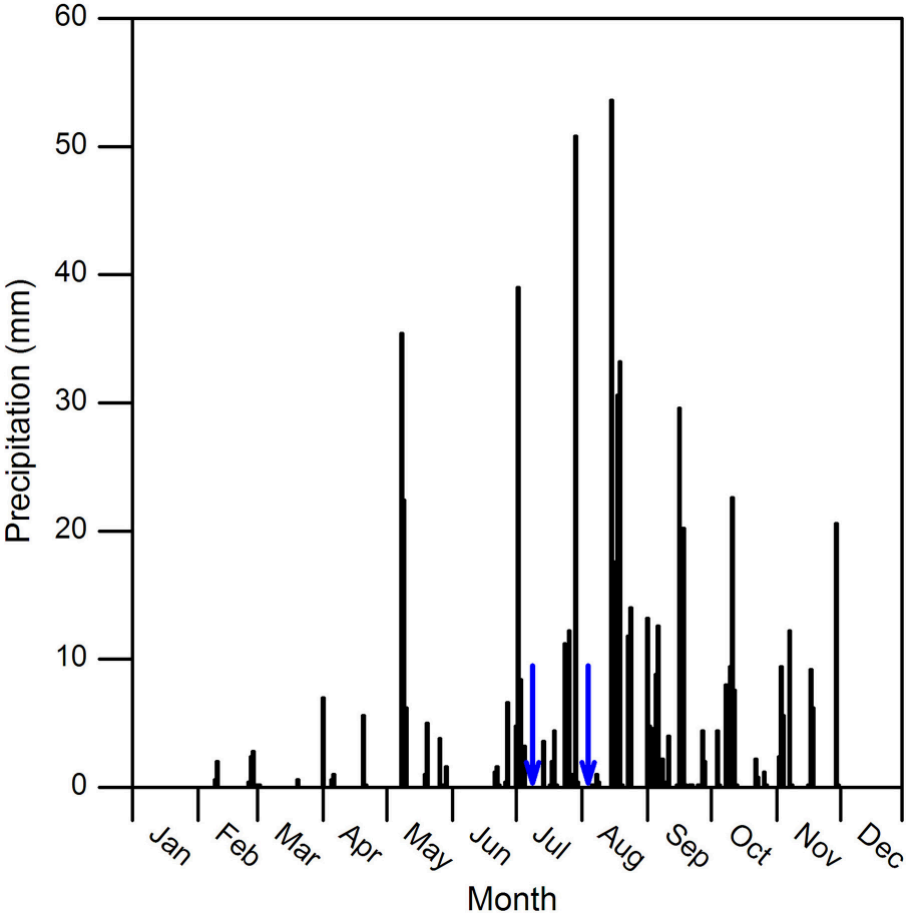
**Precipitation data recorded at the meteorological station close to the experimental area (ca. 250 m) in 2011**. Blue arrows indicate dates for watering.

**Figure 2:**
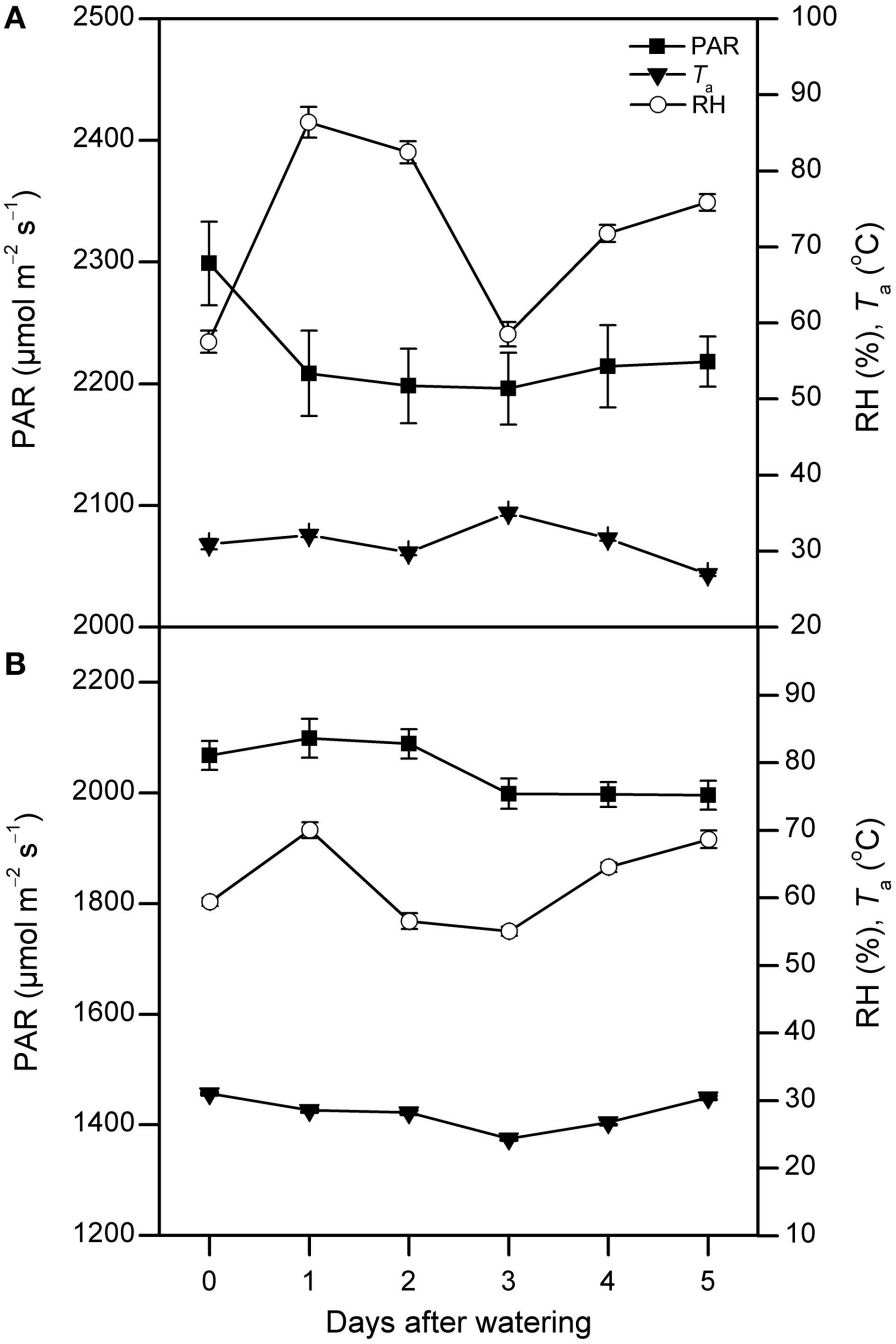
**The photosynthetically active radiation (PAR), air temperature (***T***_***a***_), and air relative humidity (RH) between 9:00 and 11:00 AM on each day following watering in July (A) and August (B), 2011 (mean ± SE, ***n*** = 9 and 15 in July and August, respectively)**. “0” stands for the day-before-watering, and the numbers “1” to “5” stand for the first to fifth day after watering.

Annual total precipitation was 663 mm in 2011. From April to October, precipitation was 588 mm and accounted for 89% of the annual total. From July to September, it was 431 mm and accounted for 65% of the total. No precipitation event greater than 5 mm was recorded during each watering treatment period (Figure 1). The instantaneous photosynthetically active radiation (PAR) value remained relatively stable, and it ranged from 2200 to 2300 μmol m^−2^ s^−1^ in July and 2000–2100 μmol m^−2^ s^−1^ in August, respectively. The air temperature (*T*_a_) was around 30°C in both experimental months. The air relative humidity (RH) increased by 50% and 26% on the first day after watering in July and August, respectively, then fell and remained nearly stable in the following days to around 50–70% in two measurement periods (Figure 2).

### Photosynthetic characteristics of co-dominant species

**Figure 3:**
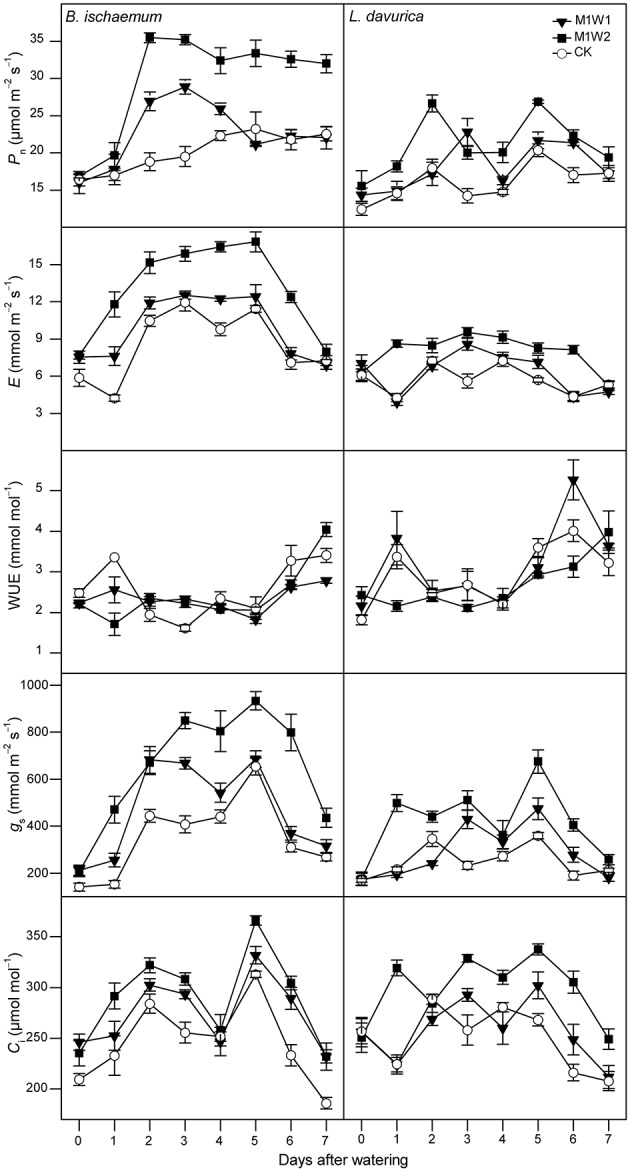
**The net photosynthetic rate (***P***_**n**_), transpiration rate (***E***), leaf instantaneous water use efficiency (WUE), stomatal conductance (***g***_**s**_), and intercellular CO_**2**_ concentration (***C***_**i**_) of ***Bothriochloa ischaemum*** and ***Lespedeza davurica*** under different treatments between 9:00 and 11:00 AM on each day following watering in July, 2011 (mean ± SE, ***n*** = 3)**. “0” stands for the day-before-watering, and the numbers “1” to “7” stand for the first to seventh day after watering.

**Figure 4:**
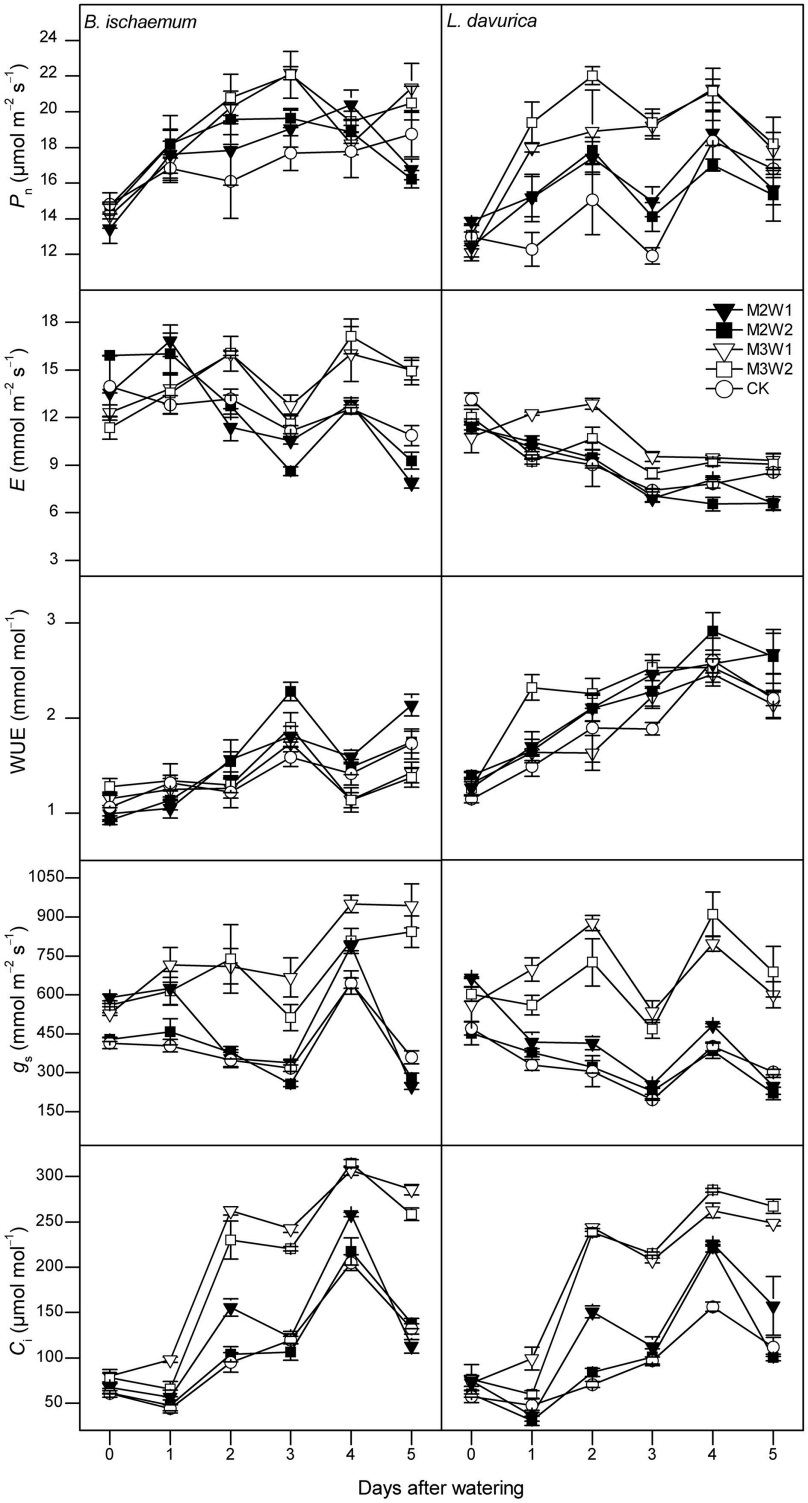
**The net photosynthetic rate (***P***_**n**_), transpiration rate (***E***), leaf instantaneous water-use efficiency (WUE), stomatal conductance (***g***_**s**_) and intercellular CO_**2**_ concentration (***C***_**i**_) of ***Bothriochloa ischaemum*** and ***Lespedeza davurica*** under different treatments between 9:00 and 11:00 AM on each day following watering in August, 2011 (mean ± SE, ***n*** = 3)**. “0” stands for the day-before-watering, and the numbers “1” to “5” stand for the first to fifth day after watering.

Before watering (day 0), there were no significant differences in all photosynthetic parameters of *B. ischaemum* or *L. davurica* across experimental plots in both months (Figures 3, 4).

**Figure 5:**
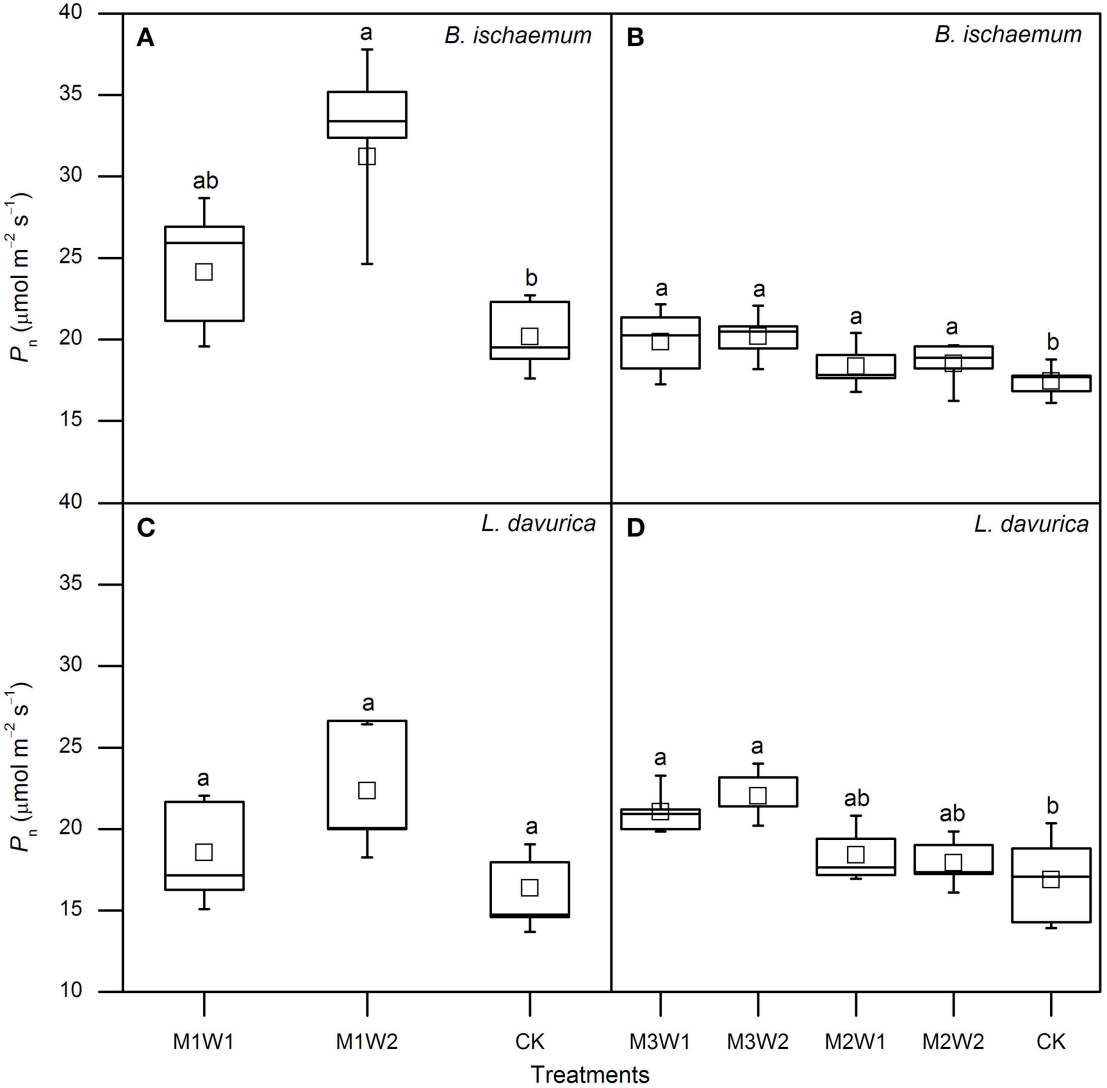
**The net photosynthetic rates (***P***_**n**_) of ***Bothriochloa ischaemum*** and ***Lespedeza davurica*** under each treatment after watering in July (A,C) and August (B,D)**. Data derived from 5 days after watering, and squares in boxes indicate averages of 5 days. The lowercase letters above boxes indicate significant differences between treatments (*P* < 0.05).

In July, after watering, photosynthetic parameters started to increase in *B. ischaemum* under both M1W1 and M1W2 treatments. The maximum increments with respect to control treatment (CK) appeared on the second to third day, and photosynthetic rates (*P*_n_) were 28.9 and 35.5 μmol m^−2^ s^−1^, with 48 and 88% increases, transpiration rates (*E*) were 7.6 and 11.8 mmol m^−2^ s^−1^, with 80 and 178% increases, stomatal conductance (*g*_*s*_) were 255.3 and 470.1 mmol m^−2^ s^−1^, with 67 and 208% increases, and intercellular CO_2_ concentrations (*C*_i_) were 293.5 and 291.6 μmol mol^−1^, with 15 and 25% increases, respectively. In *L. davurica*, all photosynthetic parameters did not increase in comparison with CK at the first 2 days under M1W1 treatment, however under M1W2 treatment, rapid increases were observed in all photosynthetic parameters except WUE, and *P*_n_ was 26.6 μmol m^−2^ s^−1^, with 48% increase compared with CK, *E* was 8.6 mmol m^−2^ s^−1^ (102% increase), *g*_s_ was 498.2 mmol m^−2^ s^−1^ (130% increase), and *C*_i_ was 319.4 μmol mol^−1^ (42% increase). Averaged values of *P*_n_ during the first 5 days after watering under M1W2 treatment were 31.2 and 22.3 μmol m^−2^ s^−1^ in *B. ischaemum* and *L. davurica*, and were about 55 and 36% higher than those under CK (20.2 and 16.4 μmol m^−2^ s^−1^), respectively (Figure 5). Averaged *P*_n_ values of M1W1 treatment were not significant different with CK in both species (Figure 5). Besides *P*_n_, the *E*, *g*_s_ and *C*_i_ values of two species also had some extent of increase after watering despite fluctuations were observed during the whole measured period (Figure 3). The leaf instantaneous water use efficiency (WUE) under M1W1 and M1W2 treatments kept stable or decreased compared with CK. For example, under M1W2 treatment, leaf WUE values decreased about 49 and 36% on the first day after watering in *B. ischaemum* and *L. davurica*, respectively (Figure 3). On the seventh day after watering, photosynthetic parameters of two species under both watering treatments decreased toward to same levels as CK, except *P*_n_ and *C*_i_ of *B. ischaemum* under M1W2 treatment (Figure 3).

In August, 5-day-averaged *P*_n_ values of two species after watering increased under all four watering treatments (i.e., M2W1, M2W2, M3W1, and M3W2). The maximum *P*_n_ values for *B. ischaemum* and *L. davurica* were 20.2 and 22.0 μmol m^−2^ s^−1^ under M3W2 treatment, and about 16 and 30% significantly higher than CK, respectively (Figure 5). The *P*_n_ values of *B. ischaemum* under all watering treatments were significantly higher than CK, while in *L. davurica*, there were only significant under M3W1 and M3W2 treatments (Figure 5). The WUE values of two species presented the similar change pattern as in July, i.e., kept stable or decreased (around 20%) on the first day after watering, except an increase was observed under M3W2 treatment in *L. davurica* (Figure 4). Under M2W1 and M2W2 treatments, the *P*_n_, *E*, *g*_s_ and *C*_i_ values of *B. ischaemum*, and *E*, *g*_s_ and *C*_i_ values of *L. davurica*, did not obviously increase during the measured period (Figure 4). Comparing with the watering experiment in July, the increments of photosynthetic parameter with respect to CK of both species in August were much lower, e.g., the *P*_n_ values increased up to 29 and 53% in *B. ischaemum* and *L. davurica* compared with 48 and 88% in July, respectively (Figures 3, 4).

### Community composition and structure

At the end of the growing season, importance value index (IVI) values of *B. ischaemum* were significantly higher under M2W2 and M3W1 treatments (*P* = 0.035 in both cases). The IVI values of *L. davurica* slightly decreased under watering treatments, while differences between all treatments were not significant (Table 2).

**Table 2:** **Importance value index (IVI) of each species in the community under each watering treatment at the end of the growing season (2 November, 2011)**.

**Species**	**Family**	**Importance value index (IVI)**						
		**CK**	**M1W1**	**M1W2**	**M2W1**	**M2W2**	**M3W1**	**M3W2**
*Artemisia argyi*	Asteraceae	0	0	0	0	13.30	0	0
*Artemisia capillaris*	Asteraceae	0	0	0	16.90	0	0	0
*Artemisia vestita*	Asteraceae	20.27	16.14	18.42	21.17	10.60	0	20.07
*Astragalus adsurgens*	Fabaceae	0	0	0	0	9.91	0	0
*Astragalus melilotoides*	Fabaceae	22.86	0	17.57	13.89	14.38	13.18	13.16
*Bothriochloa ischaemum*	Poaceae	64.89bc	72.40ab	65.24abc	66.38abc	76.02a	74.80a	60.76c
*Carduus nutans*	Asteraceae	40.99	0	13.96	19.38	8.48	12.69	0
*Cirsium setosum*	Asteraceae	34.92	18.99	23.32	18.38	33.75	24.32	25.66
*Cleistogenes caespitosa*	Poaceae	0	0	20.88	0	22.02	14.95	17.38
*Cleistogenes chinensis*	Poaceae	29.53	17.79	17.11	20.88	21.40	10.28	8.54
*Cleistogenes songorica*	Poaceae	23.85	17.06	0	0	0	0	10.26
*Clematis fruticosa*	Ranunculaceae	0	20.72	15.39	0	13.00	12.26	13.86
*Glycyrrhiza glabra*	Fabaceae	14.07	14.74	0	17.34	0	0	0
*Heteropappus altaicus*	Asteraceae	25.28	0	29.96	17.90	12.48	18.21	7.11
*Lespedeza davurica*	Fabaceae	68.69	54.95	62.48	59.57	53.28	65.82	56.62
*Leymus secalinus*	Poaceae	24.40	29.46	31.75	0	24.93	25.70	36.29
*Oxytropis bicolor*	Fabaceae	0	11.90	17.82	0	7.28	8.10	0
*Polygala tenuifolia*	Polygalaceae	12.13	10.90	21.45	31.02	17.25	0	17.61
*Potentilla tanacetifolia*	Rosaceae	0	7.18	0	0	0	10.42	0
*Salsola collina*	Chenopodiaceae	0	0	0	0	0	8.34	12.59
*Scorzonera austriaca*	Asteraceae	16.06	0	0	0	0	0	8.15
*Sonchus oleraceus*	Asteraceae	0	10.91	0	0	0	10.50	0
*Stipa bungeana*	Poaceae	28.43	37.59	42.12	51.10	35.10	40.27	35.53
*Taraxacum mongolicum*	Asteraceae	0	0	14.18	0	0	0	0
*Vicia sepium*	Fabaceae	0	0	0	0	0	12.45	33.82
*Viola philippica*	Violaceae	0	7.35	0	0	0	18.25	13.57

*Different lowercase letters indicate significant differences between treatments (P < 0.05)*.

In June, there were no significant differences in species richness (*R*), Shannon and Wiener's diversity index (*H*), Simpson diversity index (*D*), and Pielou's evenness index (*J*) throughout all treatments. In November, the *R, H*, and *D* indices under watering treatments slightly increased compared with CK, while there were still no significant differences throughout all treatments in all indices (Table 3).

**Table 3:** **Species richness (***R***), Shannon and Wiener's diversity index (***H***), Simpson diversity index (***D***), and Pielou's evenness index (***J***) of the communities before (June) and after (November) watering treatments in 2011 (mean ± SE, ***n*** = 3)**.

**Indices**	**Months**	**CK**	**M1W1**	**M1W2**	**M2W1**	**M2W2**	**M3W1**	**M3W2**
*R*	June	8.33 ± 1.45	8.33 ± 0.33	8.67 ± 1.20	7.67 ± 0.33	8.00 ± 1.00	7.67 ± 0.67	7.33 ± 0.33
	November	8.00 ± 0.58	11.00 ± 0.58	9.33 ± 0.33	9.00 ± 0.58	11.00 ± 1.00	10.33 ± 0.88	10.67 ± 0.67
*H*	June	1.90 ± 0.15	1.90 ± 0.07	1.93 ± 0.11	1.89 ± 0.02	1.93 ± 0.12	1.81 ± 0.11	1.87 ± 0.06
	November	1.91 ± 0.02	2.16 ± 0.03	2.08 ± 0.04	2.03 ± 0.06	2.15 ± 0.06	2.07 ± 0.09	2.18 ± 0.03
*D*	June	0.83 ± 0.02	0.82 ± 0.01	0.83 ± 0.01	0.83 ± 0.01	0.84 ± 0.02	0.82 ± 0.02	0.83 ± 0.01
	November	0.83 ± 0.01	0.86 ± 0.01	0.86 ± 0.01	0.85 ± 0.01	0.86 ± 0.01	0.85 ± 0.01	0.87 ± 0.01
*J*	June	0.91 ± 0.01	0.90 ± 0.02	0.90 ± 0.01	0.93 ± 0.01	0.95 ± 0.01	0.89 ± 0.02	0.94 ± 0.04
	November	0.92 ± 0.03	0.90 ± 0.01	0.93 ± 0.01	0.93 ± 0.03	0.90 ± 0.01	0.89 ± 0.01	0.92 ± 0.03

### Community above-ground biomass

**Figure 6:**
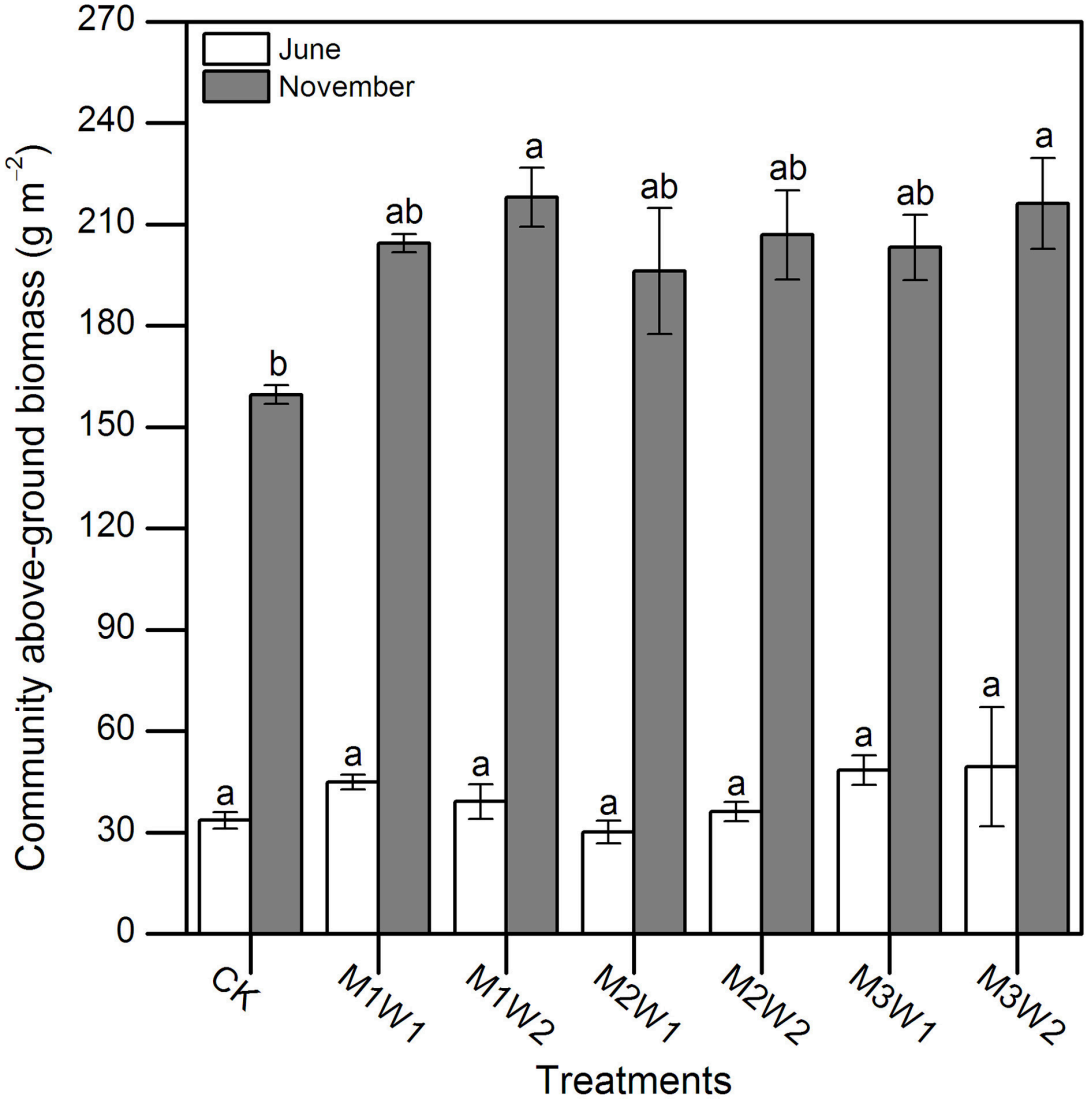
**Community above-ground biomass under each treatment in June and November (mean ± SE, ***n*** = 3)**. Different lowercase letters indicate significant differences between treatments in each month (*P* = 0.471 and 0.005 in June and December, respectively).

In June 2011, there were no significant differences in community above-ground biomasses across all plots (*P* = 0.471; Figure 6), which ranged from 30 to 50 g m^−2^. At the end of the growing season (2 November), community above-ground biomasses under M1W2 and M3W2 treatments were significantly higher than CK (*P* = 0.031 and 0.039, respectively), and increased about 37 and 35%, respectively; while differences between other treatments were not significant (*P* > 0.05, Figure 6). The highest biomass production occurred in M1W2 treatment (218.1 g m^−2^), and the lowest one was in CK treatment (159.6 g m^−2^).

## Discussion

Water availability following precipitation pulses is the critical factor that determines plant growth and physiological processes in semi-arid regions (Schwinning and Sala, 2004). In the current study, transient increase in air humidity following watering was observed, suggested that precipitation pulse events may shortly affect micro-meteorological conditions (Figure 1). Previous studies in semi-arid regions showed that soil water content increased after precipitation pulses, and positive relationships were observed between soil water content and plant photosynthesis (Xu and Li, 2006; Volder et al., 2010; Thomey et al., 2014). Unfortunately, we did not simultaneously monitor fluctuations of soil water content. In general, soil water content can be affected by soil texture, canopy interception, run-off, and evaporation after precipitation events (Loik et al., 2004). In this study, all plots have same soil type (i.e., Calcic Cambisols), and water was evenly sprinkled to community ground at 18:00 PM to avoid run-off, interception, and evaporation loss. Therefore, we inferred that water infiltration within all experimental plots should be comparable and watering treatments could improve soil water content. Consequently, significant increases of photosynthetic rates and other gas exchange parameters were observed, which could attribute to improved water availability after watering.

Our research indicated that photosynthetic rate and other parameters of two co-dominant species initially increased, and the peak values of the photosynthetic rate, transpiration rate, stomatal conductance, and intercellular CO_2_ concentration appeared on the second to third day after watering (Figures 3–5). Increases of grass photosynthesis after precipitation pulses have been reported in arid and semi-arid ecosystems (e.g., Huxman et al., 2004; Ignace et al., 2007). Rapid responses of photosynthesis were observed in various studies regardless species and site conditions (Huxman et al., 2004; Ignace et al., 2007; Resco et al., 2008; Thomey et al., 2014), while the peak values may appeared on different days, e.g., the third to fourth day in dominant species of arid and semi-arid grasslands (Thomey et al., 2014) and the seventh day in two C_4_ bunchgrasses (i.e., *Heteropogon contortus* and *Eragrostis lehmanniana*; Huxman et al., 2004; Ignace et al., 2007). These diverse photosynthetic responses could be due to effects of soil texture, plant functional types and antecedent environmental conditions (Huxman et al., 2004; Resco et al., 2009; Funk and Zachary, 2010). Our results also indicated that leaf instantaneous water use efficiency (WUE) kept stable or decreased just after watering in two co-dominant species, particularly in July, which could be explained by asymmetrical increases in transpiration and photosynthetic rates (see Figures 3, 4). Contradictory phenomenon was found in an arid sage scrub community, in which perennial shrub species (*Ricinus communis* and *Salvia mellifera*) maintained high photosynthetic rates, to increase water use efficiency rather than stomatal conductance after irrigation from short-term water stress (Funk and Zachary, 2010). Rapid increases in stomatal conductance and intercellular CO_2_ concentration after watering in the two species observed in this study indicated that both species performed effective stomatal control under favorable water conditions, which led to prompt increases in both photosynthetic and transpiration rates, and greater increase was recorded in transpiration rate, resulted in a decrease in instantaneous leaf WUE.

Plants with different photosynthetic pathways may preserve different response behaviors to precipitation pulses (Volder et al., 2010; Throop et al., 2012). C_4_ species normally have higher photosynthetic rate and WUE than C_3_ species in water limited environments, as shown in this experiment (Figure 5). Ripley et al. (2010) reported C_4_ grasses had slower recovery of photosynthesis after re-watering, comparing with C_3_ plants through increasing stomatal conductance. While in this study, we observed prompt increases of stomatal conductance in both C_4_ and C_3_ species, led to initially recovery of photosynthesis in the two species. Considering relative favorable precipitation conditions before watering in our experiment (see Figure 1), we inferred one possible explanation could be the species did not suffered from biochemical, stomatal, and mesophyll limitations under pre-watering condition that would potentially affect the recovery of photosynthesis (Flexas et al., 2008; Resco et al., 2009).

Small precipitation events may only wet upper soil, where larger events could increase soil water in deeper layer, and evaporation and vapor diffusion rates decline while rates of plant water uptake increase (Schwinning and Sala, 2004). In this study, photosynthetic parameters of the two species under larger watering quantities were higher than smaller ones, and duration of watering effect was longer (Figures 3, 4). Regarding the increments of photosynthetic parameters, our results showed that watering in July was more favorable for photosynthesis of the two species than in August, suggesting precipitation pulses during early growing season have more promoting effects than ones in the later stage, particularly in *B. ischaemum* (Figures 3–5). Similar results were described by Ignace et al. (2007) in two bunchgrasses (i.e., *H. contortus* and *E. lehmanniana*), they reported the pulse event in June (early growing season) could improve plant photosynthesis while ones in August did not result in increase in plant water status and photosynthesis. Zeppel et al. (2014) also concluded precipitation changes during dry seasons may have larger effects compared with in wet seasons, because favorable soil moisture conditions during rainy seasons could maintain high plant performance, and therefore diluted the effect of extra watering addition. Our experiment confirmed these since we recorded abundant precipitation conditions before the watering in August (143 mm in total, see Figure 1), despite similar antecedent soil water contents during 2 months (Table 1).

Precipitation is the critical determining factor for species distribution and persistence in arid and semi-arid regions (Knapp et al., 2001; Dukes et al., 2005). Changes in precipitation regimes are likely to have profound consequences for community structure and ecosystem function in arid and semi-arid ecosystems (Báez et al., 2012; Cherwin and Knapp, 2012). In this study, under watering treatments, the species important value index (IVI) of *B. ischaemum* increased, whereas that of *L. davurica* decreased at the end of the growing season, indicating that *B. ischaemum* was more affected by short-term water addition (Table 2). Research in the Inner Mongolian steppe regions and the North American grasslands suggested that, increase in mean annual precipitation or direct addition of water increased above-ground net primary productions and species richness (Harpole et al., 2007; Bai et al., 2008; Ma et al., 2010; Wilcox et al., 2015). Our study observed significant increases in community above-ground biomass following watering (Figure 6), while species diversity had no significant change after one growing season (Table 3). Considering results of IVI and photosynthetic responses in dominant species, we infer that the biomass of *B. ischaemum* will increase under the year with more precipitation pulses during the early stage of the growing season, and may outperform *L. davurica* in these years.

Changes in precipitation regimes often lead to non-linear and unexpected responses in plant communities (Harpole et al., 2007; Suttle et al., 2007), and short-term dynamics may not reflect long-term shifts in community composition (Sandel et al., 2010). The total precipitation in 2011 was higher than the long-term averaged amount (663 vs. 550 mm), which could weaken the effect of short-term watering on community characteristics (Zeppel et al., 2014). The phenology of species could potentially influence community composition and structure, even more, seasonal fluctuations in precipitation regimes may shift plant phenology (Prevéy and Seastedt, 2014). While the relation between species emerging and their phenology was not investigated in the current study. Therefore, it is necessary to conduct continuous and long-term experiments to fully understand community composition and structure dynamics in response to altered precipitation regimes in the region.

## Conclusion

The study sheds light on potential effects of precipitation pulses on natural grassland in the semi-arid Loess Plateau region. Results indicated that photo-physiological characteristics of the two co-dominant species responded significantly, and all photosynthetic parameters were initially increased following watering, while leaf water use efficiency kept stable. Data indicated that *B. ischaemum* was more sensitive in response to watering compared with *L. davurica*, in particular to watering during the early stage of the growing season, suggesting that *B. ischaemum* could outperform *L. davurica* in years with more precipitation in the early rainy season. Results also showed that watering significantly increased grassland community above-ground biomass, while species diversity did not significantly change after one growing season. All these imply that plant species-specific responses in eco-physiological traits to watering may result in changes of community composition under altered precipitation regimes, and highlight necessities to bridge eco-physiological traits in species-level to community dynamics in future studies.

## Author contributions

BX designed the study, FN and DD conducted the field work, BX, FN, DD, JC, PX, HZ, ZW, and BX were involved in data analysis and writing the paper.

### Conflict of interest statement

The authors declare that the research was conducted in the absence of any commercial or financial relationships that could be construed as a potential conflict of interest.
